# Modeling Neuroimmune Interactions in Human Subjects and Animal Models to Predict Subtype-Specific Multidrug Treatments for Gulf War Illness

**DOI:** 10.3390/ijms22168546

**Published:** 2021-08-09

**Authors:** Francisco J. Carrera Arias, Kristina Aenlle, Maria Abreu, Mary A. Holschbach, Lindsay T. Michalovicz, Kimberly A. Kelly, Nancy Klimas, James P. O’Callaghan, Travis J. A. Craddock

**Affiliations:** 1 Institute for Neuro-Immune Medicine, Nova Southeastern University, Fort Lauderdale, FL 33314, USA; fc414@mynsu.nova.edu (F.J.C.A.); kaenlle@nova.edu (K.A.); mabreu1@nova.edu (M.A.); nklimas@nova.edu (N.K.); 2 Department of Clinical Immunology, College of Osteopathic Medicine, Nova Southeastern University, Fort Lauderdale, FL 33314, USA; 3 Miami Veterans Affairs Healthcare System, Miami, FL 33125, USA; 4 Department of Psychology & Neuroscience, College of Psychology, Nova Southeastern University, Fort Lauderdale, FL 33314, USA; mholschb@nova.edu; 5 Health Effects Laboratory Division, Centers for Disease Control and Prevention, National Institute for Occupational Safety and Health, Morgantown, WV 26505, USA; yqp4@cdc.gov (L.T.M.); gup2@cdc.gov (K.A.K.); jdo5@cdc.gov (J.P.O.); 6 Department of Computer Science, College of Engineering and Computing, Nova Southeastern University, Fort Lauderdale, FL 33314, USA

**Keywords:** neuroinflammation, blood–brain barrier, logical modeling, regulatory biology, homeostatic regulation, treatment course prediction, gulf war illness, post-traumatic stress disorder, multidrug treatments, repurposing

## Abstract

Gulf War Illness (GWI) is a persistent chronic neuroinflammatory illness exacerbated by external stressors and characterized by fatigue, musculoskeletal pain, cognitive, and neurological problems linked to underlying immunological dysfunction for which there is no known treatment. As the immune system and the brain communicate through several signaling pathways, including the hypothalamic–pituitary–adrenal (HPA) axis, it underlies many of the behavioral and physiological responses to stressors via blood-borne mediators, such as cytokines, chemokines, and hormones. Signaling by these molecules is mediated by the semipermeable blood–brain barrier (BBB) made up of a monocellular layer forming an integral part of the neuroimmune axis. BBB permeability can be altered and even diminished by both external factors (e.g., chemical agents) and internal conditions (e.g., acute or chronic stress, or cross-signaling from the hypothalamic–pituitary–gonadal (HPG) axis). Such a complex network of regulatory interactions that possess feed-forward and feedback connections can have multiple response dynamics that may include several stable homeostatic states beyond normal health. Here we compare immune and hormone measures in the blood of human clinical samples and mouse models of Gulf War Illness (GWI) subtyped by exposure to traumatic stress for subtyping this complex illness. We do this via constructing a detailed logic model of HPA–HPG–Immune regulatory behavior that also considers signaling pathways across the BBB to neuronal–glial interactions within the brain. We apply conditional interactions to model the effects of changes in BBB permeability. Several stable states are identified in the system beyond typical health. Following alignment of the human and mouse blood profiles in the context of the model, mouse brain sample measures were used to infer the neuroinflammatory state in human GWI and perform treatment simulations using a genetic algorithm to optimize the Monte Carlo simulations of the putative treatment strategies aimed at returning the ill system back to health. We identify several ideal multi-intervention strategies and potential drug candidates that may be used to treat chronic neuroinflammation in GWI.

## 1. Introduction

Gulf War Illness (GWI) is a chronic disorder that affects up to one-third of the 700,000 veterans of the Persian Gulf War of 1991. Many symptoms of GWI, including fatigue, musculoskeletal pain, cognitive, and neurological problems [1], are indicative of a neuroinflammatory disorder, and abnormal regulation of the hypothalamic–pituitary–adrenal (HPA) axis, a key component in the body’s stress response, has been associated with this chronic condition [2,3,4,5,6]. Yet, the cause and underlying mechanisms of GWI are still relatively unknown, and there is no known treatment. Progress needs to be made to provide relief to this aging population, but the heterogeneity of the illness presentation provides complications. Subtyping the illness is one option to improve diagnostics and identify potential subtype-specific treatment avenues.

Possible explanations for the cause of GWI include exposure to mild traumatic brain injury and chemical/biological weapons [7] as well as the involvement of a neuroinflammatory signaling cascade triggered by exposure to a neurotoxin (e.g., the organophosphate sarin) and exposure to a combat environment [8,9,10]. Genetic susceptibility [11,12] of veterans may further contribute to the resulting changes in brain function observed in GWI [13,14,15]. Furthermore, confounding and/or exacerbating factors often complicate this diagnosis. Veterans suffering from GWI are often also diagnosed with post-traumatic stress disorder (PTSD) [16,17]. A population-based study estimates that 35% of veterans with GWI also suffer from PTSD [18], which exacerbates all the overall clinical symptoms of GWI [19]. As there is a substantial overlap of PTSD symptoms with the symptoms defining GWI [20], with both PTSD [21,22,23,24] and GWI [6,25,26] exhibiting immunological dysfunction and abnormal neural synchrony [14], this suggests that GWI and PTSD share several core pathophysiological processes and that these processes may be altered in the context of co-morbidity. As such, the presence or absence of PTSD in GWI provides one mean to subtype this illness. 

While the immune system, as an independent agent, provides the human body with a defense system that protects against foreign proteins and chemicals, it cannot be considered independent from the body as a whole, or the brain in particular. The brain plays a critical role in the regulation and modulation of immunity, effectively integrating the nervous and immune systems, the two most complex systems of the body for the maintenance of homeostasis, into one. The study of these neuroimmune interactions along with the endocrine system and resulting behaviors is collectively known as psychoneuroimmunology and studying the functional significance of the relationships among these systems is the focus of the field. Analyzing the mechanisms that functionally alter this single overarching system can lead to a significantly enhanced understanding of the unique and combined contributions of hormones, the brain, behavior, and the immune system to health and disease.

The immune system and the brain communicate through several signaling pathways. Two of the major pathways involved in this crosstalk are the sympathetic nervous system via the sympathetic–adrenal–medullary axis and the hypothalamic–pituitary–adrenal (HPA) axis. Both release blood-borne mediators, including cytokines, chemokines, and hormones, which are then subject to further regulation. The brain and spinal cord, which comprise the central nervous system (CNS), are kept isolated from signaling in the blood flow of the peripheral body by a monocellular interface called the blood–brain barrier (BBB). This division is made possible by several parallel barriers that include most notably the capillary bed of the CNS and the choroid plexus. These barriers at one level create the dichotomy between the circulating factors of the immune system and the components of the CNS only to regulate the interactions between the immune system and the CNS at other levels. The BBB is thus an integral part of the neuroimmune axis. Many factors, including stress, regulate the permeability of the BBB. Corticotropin-releasing hormone (CRH), released by the HPA axis during times of stress, has proinflammatory effects mediated through mast cells, which regulate BBB permeability [27,28]. Furthermore, CRH release leads to the secretion of catecholamines and glucocorticoids, which exert their effects on the BBB. Exposure to acute or chronic stress activates the HPA axis, whether the stressor is mental or physiological distress, even inducing, and can lead to BBB breakdown [27,28].

Not only is the HPA axis activated by stress, HPA activity is also tied to both the hypothalamic–pituitary–gonadal (HPG) axis and the immune system, among others, and is further influenced by brain signaling outside the hypothalamus. Moreover, weakening the BBB can lead to additional or enhanced interactions among these and other systems. This level of integrated connectivity is core to the body’s adaptive capability and contributes to its resilience and vulnerabilities. As the HPA axis plays a crucial role in the body’s response to stress [29,30,31] and is one of the major information pathways connecting the peripheral systems to the CNS, several mathematical models of HPA dynamics have been proposed to understand its function [32,33,34,35,36,37]. Few models account for its detailed feed-forward regulation [37], but this is highly relevant as signaling networks such as the HPA axis do not work in isolation. Even fewer account for its connection to other bodily systems.

A complex network of regulatory interactions that possess feed-forward and feedback connections, such as the aforementioned neuro-immune axis, can have multiple response dynamics that may include several stable homeostatic states beyond a normal healthy state [38,39,40,41,42,43,44,45,46]. Due to homeostatic feedback mechanisms, acute or minor changes to this system return to the baseline, or a normal healthy state. Nevertheless, under more severe perturbations, the system may shift from a normal homeostatic state to a new stable state. This disturbance may consist of a single event or an ongoing external insult that further changes the underlying dynamics. We believe that these regulatory alterations are a key cause of the resistance to therapy seen in neuroinflammatory illnesses and may explain some of the hardships in attaining lasting remission. By identifying and detailing these alternative homeostatic regimes, it may be possible to design treatments aimed at returning the overall system back to its original healthy state.

In this work, we propose to build on our ongoing research directed at mapping complex inflammatory mechanisms in GWI to improve our understanding of the immunologic underpinnings of GWI, the compounding effects of co-morbidity with PTSD, and the potential of this comorbidity to define a unique subtype of GWI. Furthermore, we propose to take advantage of our ongoing work in predictive modeling to assess possible changes to putative treatments of GWI in the context of a probable PTSD diagnosis or lack thereof. We do this by constructing a logic model representation of the neuroimmune system across the BBB connecting the CNS to the peripheral immune system. As we cannot assess the neuroimmune state in the brain of living GWI subjects, we topologically compare the model results of a GWI cohort with and without PTSD symptoms to mouse models of GWI in blood and use the corresponding neuroimmune states in mouse brain to infer the blood–brain state of the GWI subjects. This inference is then used to guide predictive modeling of treatment courses designed to return the overall neuroimmune system to healthy regulation. 

## 2. Results

### 2.1. The GWI Cohort

The total cohort used in this study was comprised of 89 male veterans recruited through the Miami Veterans Affairs (VA) Medical Health Center. The average age of the cohort was 43.68 ± 7.42 years with an average BMI of 29.49 ± 5.42. The sample consisted of 2.2% Asian, 22.5% Black, 46.1% White Hispanic, 28.1% White, and 1.1% Other. The full demographics are presented in Table 1. 

The full sample included *n*  =  51 male veterans meeting the criteria for GWI in addition to *n*  =  38 healthy sedentary veteran controls (HC). McDonald et al. (2014) [47] examined the diagnostic accuracy of applying a cut score method to the Davidson Trauma Score (DTS) in a population of U.S. military veterans and found that using a cut score of 70 for the total DTS score offered optimal diagnostic accuracy, correctly classifying 90% of cases and providing an accurate estimate of PTSD population prevalence. Of the 51 GWI subjects, *n* = 32 had a total DTS of 70 or greater. The group of GWI subjects with DTS scores above 70 are considered to have a high probability of having PTSD and are denoted as GWI_H_, while the remainder have a low probability of having PTSD and labelled as GWI_L_. 

Statistical comparisons (Table 1) were made between both the total GWI and HC groups (p_2_) as well as between the GWI_H_, GWI_L_, and HC groups (p_3_), using ANOVA for the continuous variables and the χ^2^ test for categorical variables. No statistical differences were found in age, BMI, racial representation, marital status, employment status, or average number of years in school. 

**Figure 1:**
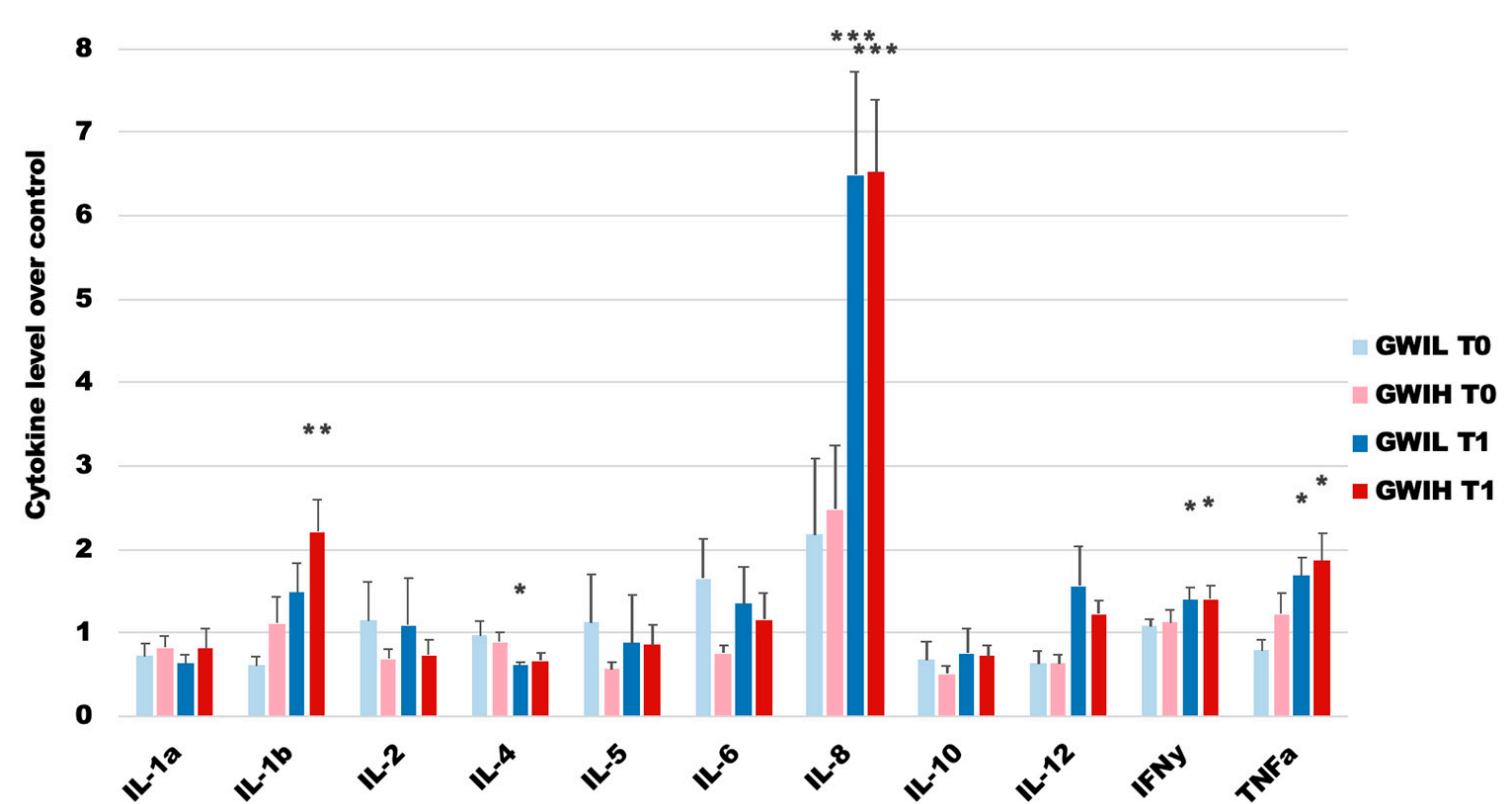
Human blood cytokine levels in PTSD-based GWI subtypes as compared to HC at rest (T0) and peak exercise during a maximal graded exercise challenge (T1). Individual cytokine values are normalized to the HC values, and bars represent the mean ratios ± SEM for each cytokine. Significance was set at * *p* < 0.05, ** *p* < 0.01, and *** *p* < 0.001 as compared to HC. The false discovery rate, as calculated via Storey et al. [48], was < 0.1 for all significant measures. Values are found in Table S1.

Blood plasma cytokine levels for the GWI subgroups at rest (T0) and peak effort in a maximal graded exercise test (T1) as compared to HC at rest are presented in Figure 1. Statistical comparisons were made using heteroscedastic two-tailed *t*-tests between the GWI subgroups and HC at both timepoints, and between the GWI subgroups within each timepoint. While there appears to be trends of immune dysregulation in the GWI subgroups at rest, there were no statistical differences between the GWI subgroups and HC or between the two subgroup conditions, owing to the large degree of variability within the measures. After immune stimulation via the graded exercise challenge the immune profiles of the GWI subgroups again show trends of immune dysregulation with several statistically significant differences, namely, elevated IL-8, IFN-γ, and TNFα in both subgroups, depressed IL-4 in GWI_L_, and elevated IL-1β in GWI_H_. Again, owing to the large degree of variability in the measures, no significant differences were found between the GWI subgroups at T1. 

### 2.2. A Long-Term Mouse Model of Gulf War Illness

**Figure 2:**
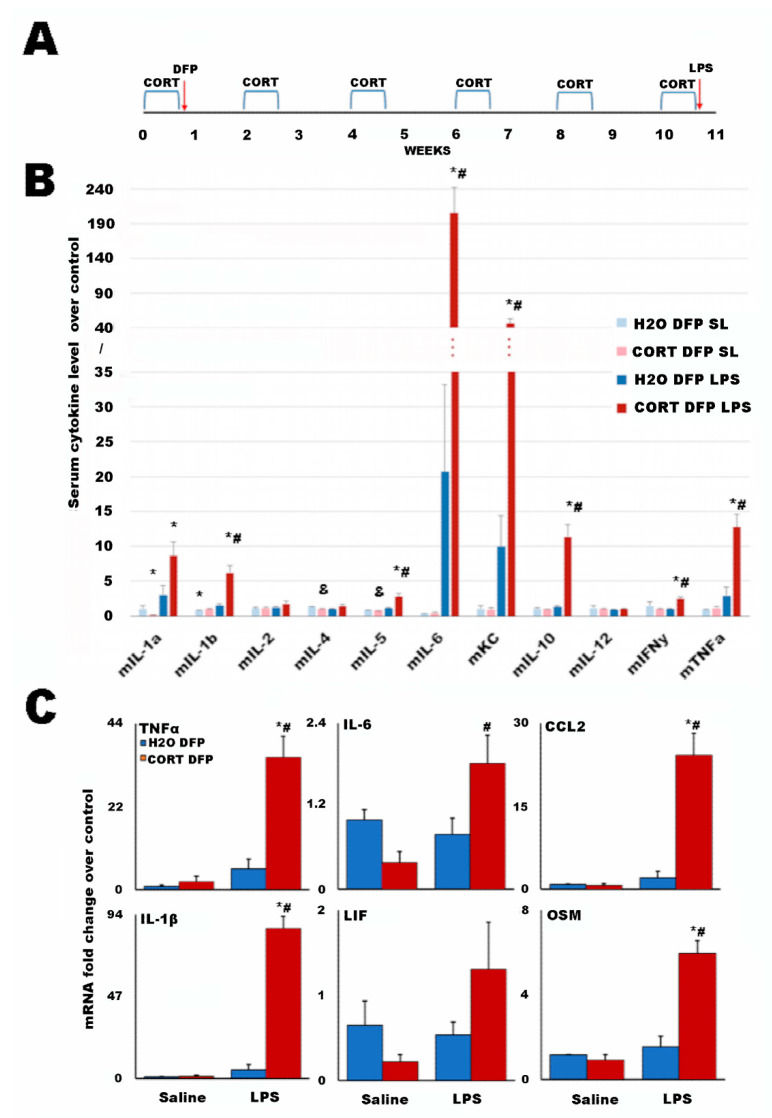
Cytokine expression in a long-term mouse model of Gulf War Illness. (**A**) Graphical representation of the animal dosing paradigm. Mice were exposed to corticosterone (CORT; 200 mg/L in 0.6% ethanol) for 4 days at a time every other week for a total of 11 weeks. DFP (4 mg/kg, i.p.) was given on Day 5 and the LPS challenge (0.5 mg/kg, s.c.) was given after the final week of CORT. (**B**) Individual serum cytokine values normalized to the water (H2O), saline solution (SL), and SL condition values. Bars represent the mean ratios ± SEM for each cytokine. Significance was set at *p* < 0.05 compared to H2O SL SL (*), and within challenge groups (&: H2O DFP SL vs. H2O DFP LPS; and #: CORT DFP SL vs. CORT DFP LPS). The false discovery rate, as calculated via Storey et al. [48], was <0.1 for all significant measures. (**C**) Cytokine mRNA was measured in the frontal cortex (*n* = 2–5/group) by qRT-PCR 6 h following exposure to LPS. Bars represent the mean ± SEM. Significance was set at *p* ≤ 0.05 for comparisons within challenge group (*: DFP vs. CORT DFP) or across challenge group (#: SL vs. LPS). Values for (**B**,**C**) are found in Tables S2 and S3, respectively.

Veterans with GWI had the potential to be exposed to several toxicants during their deployment, as well as to adverse environmental conditions [49]. Previously, a GWI animal model was established that attempted to capture the concomitant exposure to neurotoxic agents and high physiological stress, such as excessive heat and exercise, that was likely experienced during deployment by many of the veterans with GWI. By combining exposure to the sarin nerve agent surrogate diisopropyl fluorophosphate (DFP) with prior subchronic exposure to the stress hormone corticosterone (CORT), it was shown that these exposures produce significantly exacerbated neuroinflammation and pain responses, as well as changes in the structure and cellular biology of the brain and glia [50,51,52,53,54,55,56,57,58,59,60,61]. While these results have been found acutely following exposure, they have begun to be supported in current evaluations of veterans with GWI by various imaging methods, finding evidence of both neuroinflammation [13] and brain structural changes indicative of the restructuring of the glia [62]. As such, it was important to investigate the impacts of these initial CORT and DFP exposures at longer-term time points that are more relevant to the current condition of veterans with GWI. Thus, to further represent the protracted nature of GWI (i.e., 30 years since the initiating event), mice were aged for a total of 11 weeks with either H_2_O or repeated exposure to 4-day bouts of CORT every other week to reflect ongoing stress, with the potential to be representative of PTSD, following the initial exposure to CORT and DFP as utilized in previous studies (Figure 2A) [50,51,52,53,54,55,56,57,58,59,60,61]. Following this, a lipopolysaccharide (LPS) challenge was used to stimulate the immune system in lieu of exercise. To gauge the effect of the DFP exposure and LPS challenge, saline solution (SL) was used as a control. This resulted in five mouse groups analogous to the human cohort (H2O SL SL = HC; H_2_O DFP SL = GWI_L_ T0; CORT DFP SL = GWI_H_ T0; H_2_O DFP LPS = GWI_L_ T1; and CORT DFP LPS = GWI_H_ T1).

Serum cytokine levels for the GWI mouse model groups as compared to the H_2_O SL SL control are presented in Figure 2B. Statistical comparisons were made using heteroscedastic two-tailed *t*-tests between all GWI mouse model groups and the H_2_O SL SL control for both rest and LPS immune challenge timepoints, and within challenge groups for the H2O and CORT conditions. While the majority of measures for the unchallenged groups show no significant differences, there are statistical differences between the unchallenged groups and the control for IL-1α and IL-1β, and between the unchallenged groups for IL-4 and IL-5. 

The starkest differences are for the CORT DFP LPS group compared to the control and the non-stressed H_2_O DFP LPS group for IL-1α, IL-1β, IL-5, IL-6, KC (mouse equivalent of IL-8), mIL-10, IFNγ, and TNFα. While trends appear for mIL-6 and KC in the H2O DFP LPS group to be increased over the control, statistical difference is not reached due to large variance and a small group size.

Brain cytokine mRNA levels for the GWI mouse model groups as compared to the H_2_O SL SL control are presented in Figure 2C. Here, again, it is seen that the CORT DFP LPS group has statistically higher amounts of the proinflammatory cytokines IL-1β, IL-6, and TNFα than both the control and the other mouse groups. Additionally, this group also has elevated markers for microglial activation C-C Motif Chemokine Ligand 2 (CCL2) and oncostatin M (OSM). No other measures showed significant increases from the control or within groups. 

### 2.3. Male BBB Neuro-Endrocrine-Immune Model

**Figure 3:**
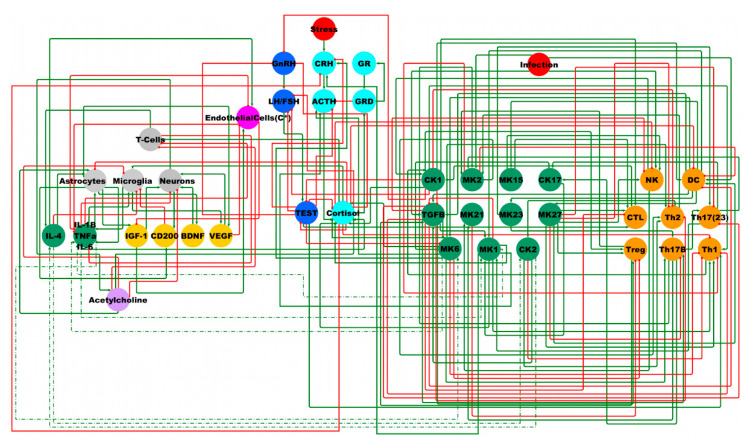
Theoretical male neuroimmune signaling network. Light blue nodes denote the HPA axis model described in [37]. Dark blue nodes denote the male HPG axis as described in [38]. Orange and green nodes on the right denote a system of immune signaling molecules and immune cells, respectively, as originally described in [39]. Grey, yellow, pink, purple, and green nodes on the left denote neuronal–glia interactions, growth factors, blood–brain barrier, neurotransmitter, and neuroimmune signaling molecules, respectively, as originally described in [53]. Red nodes denote external influences on the system. Green edges are stimulatory, and red edges are inhibitory.

Based on our previous work, we have combined a simple model of the overarching HPA–HPG–Immune system [38] with a more complex description of the immune function [39] and a brain compartment describing the neuroimmune interaction of the neurons and glia [53] (Figure 3).

**Figure 4:**
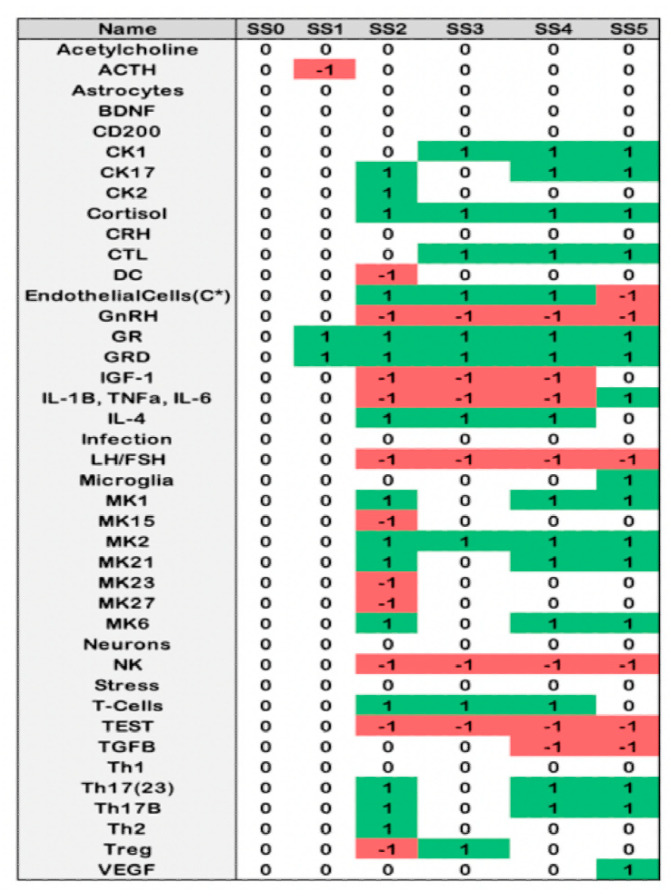
Predicted stable states of the theoretical male neuroimmune signaling network presented in Figure 3. White fill indicates a nominal state the same as the healthy control (0); green a state higher than the healthy control (1); and red state lower than healthy control (-1).

Using ternary logic modelling and performing Monte Carlo simulations of state evolution in this theoretical male neuroimmune signaling network identified six potential stable behaviors available to the system (Figure 4). The reference stable state (SS0) is that of typical health in which all measures are nominally the same as the healthy control condition. The first alternate stable state (SS1) displays a low adrenocorticotropic hormone (ACTH) concentration with a high glucocorticoid receptor number in both its single membrane bound (GR) and internalized dimerized (GRD) forms. 

The remaining alternate states (SS2, SS3, SS4, and SS5) all present with elevated HPA axis activity: cortisol, GR, and GRD, and depressed HPG activity: testosterone (TEST), luteinizing hormone (LH)/follicle-stimulating hormone (FSH), gonadotropin releasing hormone (GnRH) in addition to depressed natural killer cell activity (NK), and an increase in monokine (MK) 2 activity (IL-10, IL-22). Beyond this, the second alternate state (SS2) is distinguished by a periphery marked with elevated chemokines (CK) 2 (interleukin (IL)-4, IL-5, IL-13), CK17 (IL-17A, IL-17F), MK1 (IL-1α, IL-1β, IL-8, IL-12), MK6 (IL-6), MK 21 (IL-21), T-helper (Th) 2 cells, Th17B and Th17(23), and depressed MK15 (IL-15), MK23 (IL-23), MK27 (IL-27), dendritic cells (DC), and T regulatory (Treg) cells. In the brain compartment, SS2 presents elevated endothelial cell activity, increased T-cell activity, IL-4, and decreased pro-inflammatory cytokines (IL-1β, IL-6, TNFα), and insulin-like growth factor 1 (IGF-1). The third alternate state (SS3) presents in the periphery with elevated CK1 (IL-2, IFNγ, TNFα, TNFβ), cytotoxic lymphocytes (CTL), MK2, and Tregs, and in the brain with elevated endothelial cell activity, pro-inflammatory cytokines, and T cell activity, and depressed IL-4 and IGF-1. The fourth alternate state (SS4) likewise has elevated CK1, CTL, MK2 endothelial cell activity in the periphery, and elevated IL-4 and T-Cells in the brain. Additionally, SS4 displays elevated CK17, MK1, MK6, MK21, Th17B, Th17(23), and depressed transforming growth factor β (TGFβ) and Th1 in the periphery. The final alternate state (SS5) is marked by a periphery with elevated CK1, CK17, MK1, MK2, MK6, MK15, CTL, Th17B, and Th17(23), and in the brain with elevated pro-inflammatory cytokines, microglia activity, and vascular endothelial growth factor (VEGF), and depressed endothelial cell function.

### 2.4. Alignment of the Blood Profiles with the Model-Predicted States

**Figure 5:**
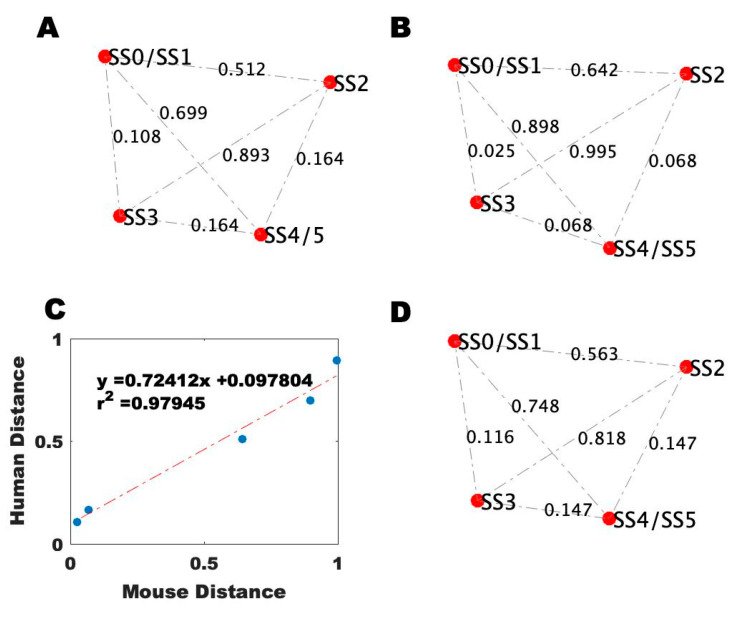
Reference blood profile topological network. (**A**) Topology of the distance between the simulated stable states using Brown’s method based on human healthy control data. (**B**) Topology of the distance between the simulated stable states using Brown’s method based on data from mice receiving a course of pure drinking water, an initial saline injection, and a follow-up injection of saline. (**C**) Regression of the human topology onto the mouse topology. (**D**) Topology of the distance between the simulated stable states using Brown’s method based on data from mice receiving a course of pure drinking water, an initial saline injection, and a follow-up injection of saline scaled to the human topology.

To determine which of the animal model conditions best represents the GWI cohort subgroups, to ultimately infer the brain state the GWI cohort subgroups, the above model describing the known interactions of the neuroimmune system was used to provide a context for comparisons between human and animal data. To do this, each subgroup condition in both the human and animal groups was compared to their respective control condition to determine the degree of agreement with each stable behavior predicted by the model (Figure 4). Initially, blood profiles for the baseline clinical data obtained from healthy control patients and data for aged mice receiving a course of pure drinking water, an initial saline injection, and a follow-up injection of saline were used to define the base topology of the simulated stable states using Brown’s method, as described in the Materials and Methods section (Figure 5). As can be seen from Figure 5A,B, the overall topology of the stable state locations is retained between humans and mice, as evidenced by the relative spacing between points. However, there is a discrepancy in the actual distance values between the stable points, owing to the difference in the human and mouse systems. By comparing the edge-by-edge distances between these networks, a strong linear correlation between mouse and human data is revealed, which is used to scale the mouse network data to humans for further analysis (Figure 5C,D). 

**Figure 6:**
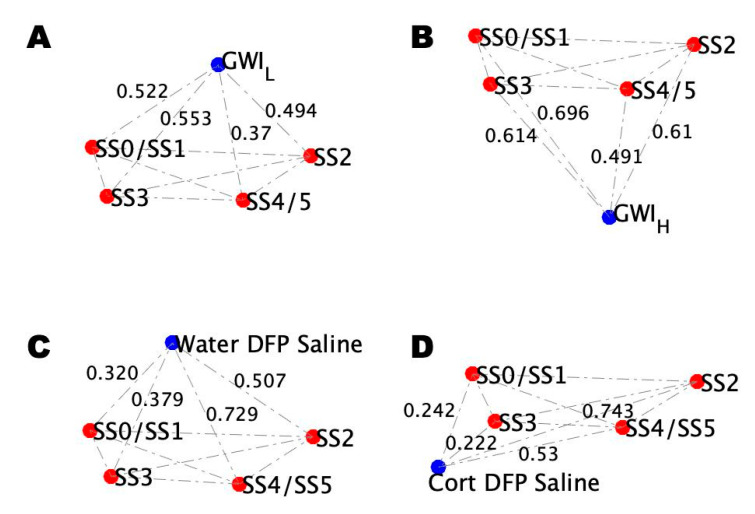
Comparison of the GWI trauma subtype blood profile topologies with mouse models at rest. (**A**) Topology of the distance between the simulated stable states for GWI_L_ at rest. (**B**) Topology of the distance between the simulated stable states for GWI_H_ at rest. (**C**) Topology of the distance between the simulated stable states from mice receiving a course of pure drinking water, an injection of DFP, and a follow-up injection of saline. (**D**) Topology of the distance between the simulated stable states from mice receiving a course of drinking water with CORT, an injection of DFP, and a follow-up injection of saline.

Cohort and mouse model subgroup condition blood profiles at rest were then added to the reference blood profile topological networks (Figure 6). The topological profile of the resulting network for GWI_H_ (Figure 6B) differed from GWI_L_ (Figure 6A) with an RMSD of 0.42. Similar topological differences are seen for DFP-exposed mice (Figure 6D) with and without prior treatment with CORT (Figure 6C). The difference between these mice conditions is comparable to the human conditions given by an RMSD of 0.33. 

Comparison of the GWI illness conditions to the mouse conditions at rest show that the GWI_L_ condition is most closely matched by the non-CORT-treated mouse model (RMSD = 0.06) rather than the CORT-treated model (RMSD = 0.36). The opposite was shown for the GWI_H_ condition, with the CORT-treated model (RMSD = 0.28) being a better match over the non-CORT-treated model (RMSD = 0.47). However, despite this agreement between the trauma/stressed states, there are noticeable differences that account for the non-zero RMSD. Both GWI illness subtypes show the closest alignment with stable state SS4/SS5. This difference is driven by GWI_H_ having a large distance from SS0/SS1 and closer alignment to SS3, while this is reversed for GWI_L_. 

This is not the case for the mouse models as the CORT-treated model shows the closest alignment with SS3, while the non-CORT-treated model shows the closest alignment with SS0/SS1. Yet, while this is a noticeable difference, the relationship between the illness condition and all four stable states determines its overall placement in the landscape and the resulting topology.

**Figure 7:**
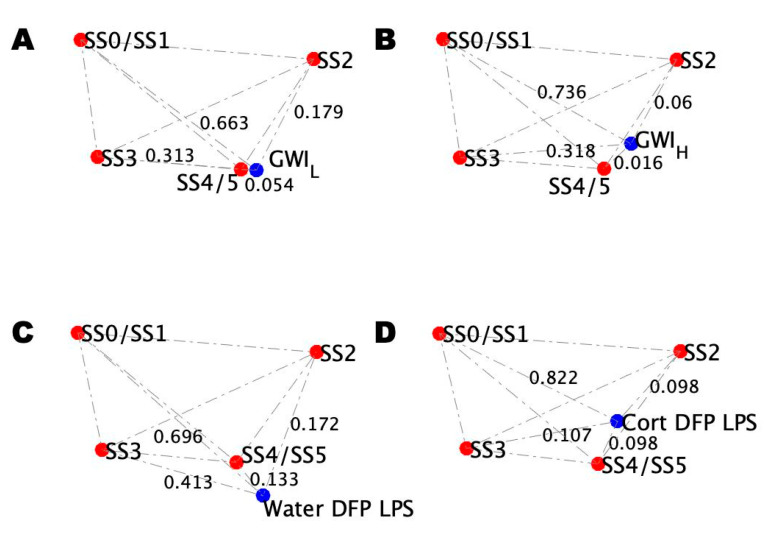
Comparison of the GWI trauma subtype blood profile topologies with mouse models under challenge. (**A**) Topology of the distance between the simulated stable states for GWI_L_ at peak exercise. (**B**) Topology of the distance between the simulated stable states for GWI_H_ at peak exercise. (**C**) Topology of the distance between the simulated stable states of mice receiving a course of pure drinking water, an injection of DFP, and a follow-up injection of LPS. (**D**) Topology of the distance between the simulated stable states of mice receiving a course of drinking water with CORT, an injection of DFP, and a follow-up injection of LPS.

Blood profiles for the model and illness conditions under challenge were separately added to the healthy blood profile topological networks (Figure 7). Unlike the at-rest condition, the non-trauma GWI_L_ and trauma GWI_H_ under challenge conditions (Figure 7A,B, respectively) present with very similar topologies (RMSD = 0.02), as do the non-CORT- and CORT-treated DFP LPS mouse models, although to a lesser degree (RMSD = 0.05). Comparison of GWI illness conditions to the mouse conditions under challenge shows that the GWI_L_ condition is still most closely matched by the non-CORT treated mouse model (RMSD = 0.02) (Figure 7C) rather than the CORT-treated model (RMSD = 0.03) (Figure 7D). However, the degree of difference is much less. Again, the GWI_H_ condition showed a better match with the CORT-treated model (RMSD = 0.02), being a better match over the non-CORT-treated model (RMSD = 0.04), again with smaller differences. Unlike the at-rest conditions, the agreements between the trauma/stressed states share the same ordering of differences between the stable state positions with only minor differences to account for the small non-zero RMSDs. In all cases, the conditions show the closest alignment with stable state SS4/SS5, followed by SS2, SS3, and finally SS0/SS1.

**Figure 8:**
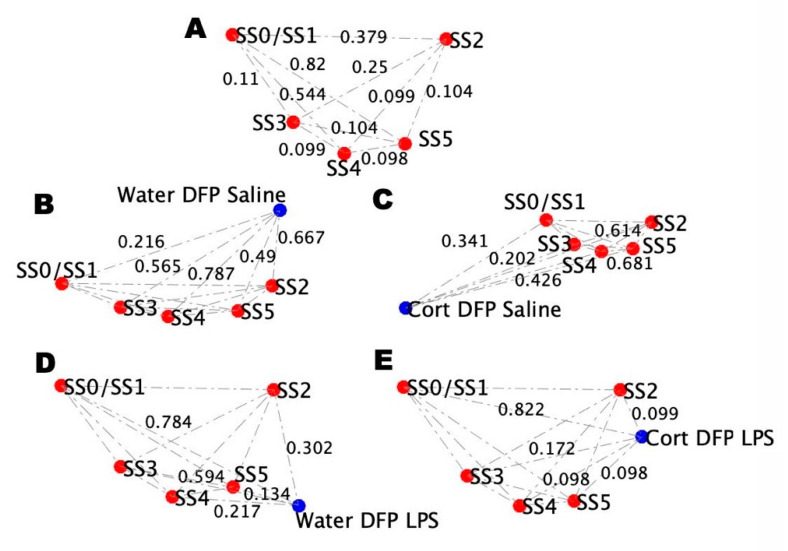
Comparison of the mouse model conditions brain and blood profile topologies. (**A**) Topology of the distance between the simulated stable states using Brown’s method based on data from mice receiving a course of pure drinking water, an initial saline injection, and a follow-up injection of saline. (**B**) Topology of the distance between the simulated stable states from mice receiving a course of pure drinking water, an injection of DFP, and a follow-up injection of saline. (**C**) Topology of the distance between the simulated stable states from mice receiving a course of drinking water with CORT, an injection of DFP, and a follow-up injection of saline. (**D**) Topology of the distance between the simulated stable states of mice receiving a course of pure drinking water, an injection of DFP, and a follow-up injection of LPS. (**E**) Topology of the distance between the simulated stable states of mice receiving a course of drinking water with CORT, an injection of DFP, and a follow-up injection of LPS.

As it is not possible to assess brain measures in the human cohort directly, adding brain measures from the mouse models to their blood profiles allows for the discrimination between SS4 and SS5 while retaining the overall structure observed in the reference brain and blood profiles (Figure 8A), and therefore infer the probable brain states for the human cohort groups. Topologically, the non-challenged mouse model, stressed and unstressed, resembles the blood-only profiles, as do the challenged stressed and unstressed conditions. For the non-challenged conditions (Figure 8B,C), the Water DFP SL mouse model retains its closest proximity to SS0/SS1, while the CORT DFP SL mouse model retains closest proximity to SS3. For the challenged conditions (Figure 8D,E), the Water DFP LPS mouse model is most comparable to the SS5 state, while the CORT DFP LPS mouse model is equidistant from both SS4 and SS5, with only a slightly longer distance to SS2. The remainder of the edge orders for all models is also retained from the blood-only comparisons (Figure 6C,D and Figure 7C,D), save for SS5. 

### 2.5. Simulated Treatment Courses

As determined above, the GWI blood profiles both align more closely with the SS4/SS5 attractor; however, the blood profiles alone cannot discriminate between these two attractor states. The alignment of the mouse profiles, GWI_L_, and GWI_H_ are best represented by the Water-DFP and CORT-DFP models, respectively, both at rest (saline) and under LPS challenge. The brain profiles of these mouse models suggest that GWI_L_ orbits SS5, while GWI_H_ orbits equidistant from states SS4 and SS5 closely followed by SS2, all of which are taken as starting points for the simulation of the treatments. Treatments were simulated, allowing for increases in cortisol, ACTH, and testosterone, inhibition of glucocorticoid receptors, and bidirectional modulation of all peripheral cytokines. 

For the SS5 start state, a minimum of three treatment targets were needed to obtain at least a 1% return to health (Table 2). The treatment course consisted of an initial phase of increasing testosterone, followed by an inhibition of CK1 cytokines, including IL-2, IFNγ, TNFα, and TNFβ, followed by inhibiting the glucocorticoid receptor with some overlap between these phases. This minimum intervention resulted in a 37% return to health.

For the SS4 start state, again, a minimum of three treatment targets were needed to obtain at least 1% return to health (Table 3). The treatment course consisted of a continual treatment of inhibiting the pro-inflammatory cytokine IL-6, which contained an initial phase of inhibiting CK1 cytokines including IL-2, IFNγ, TNFα, and TNFβ, followed by a phase of inhibiting the glucocorticoid receptor with no overlap between these two contained phases. This minimum intervention resulted in an improved 61% return to health over the SS4 prescribed treatment course.

Finally, for the SS2 start state, only two interventions were needed to obtain at least a 1% return to health (Table 4). This two-intervention treatment course consisted of a near overlapping continual treatment of inhibiting pro-inflammatory cytokine IL-6 with inhibition of the glucocorticoid receptor. This dual combination strategy resulted in a 31% return to health, less than both the SS4 and SS5 three-target treatments, but with less intervention overall.

## 3. Discussion

The wide range of symptoms observed in GWI is consistent with underlying neuroinflammatory processes. Ultimately, these processes have effects beyond the CNS, affecting immune and endocrine function in the periphery and ultimately giving rise to the symptom presentations in the illness. As the presence of PTSD symptoms exacerbates the overall clinical symptoms of GWI [19], and PTSD shows its immunological dysfunction [21,22,23,24], it is reasonable to assume that past trauma may be used to delineate two biological GWI subgroups. In the context of the neuroimmune model presented here, the high and low trauma GWI group blood profiles at rest share the closest proximity with the same altered regulatory state marked by elevated HPA axis activity, depressed HPG activity, depressed NK activity, and increased MK2, CK1, CK17, MK1, MK2, MK6, MK15, CTL, Th17B, and Th17(23) activity, albeit not with complete agreement. Under exercise challenge, these two groups converge to this common point and are nearly identical. 

These results alone suggest that the absence and presence of PTSD do not delineate specific biological subtypes of GWI. However, when projected into the neuroimmune model state space, these two subtypes present as topologically distinct at rest, suggesting that the underlying biological regulation between these two groups are unique. These modeling results are consistent with past research into the biomarkers/biosignatures of GWI. The difference in biological profiles between GWI and the controls has been shown to be more distinct and consistent under an exercise challenge [6]. At rest, GWI profiles are variable and sometimes present with inconsistent results [63,64]. Past biomarker/biosignature studies have not grouped GWI in terms of prior trauma; as such, the distinct GWI_L_ and GWI_H_ subgroups intermixed at rest would be expected to yield mixed and inconsistent results compared to controls as the proportion of each group varies. However, under exercise challenge, as the profiles of GWI_L_ and GWI_H_ converge, the typical neuro-endocrine immune signature emerges as different from the control. 

The difference between the profiles of GWI_L_ and GWI_H_ at rest is due to their relation to the remaining stable regulatory patterns that define their difference in blood alone. As neither of the subgroups aligns completely with any of the defined stable states, there are factors not accounted for in the model to hold the subgroups at their baseline positions. This may be in both the periphery or in the brain portions of the network model. Further refinement of the granularity of the peripheral endocrine and immune system elements is thus hypothesized to further define these two subgroups in human subjects. 

The topological alignment between the GWI subtypes and the animal models suggests that the use of corticosterone priming of the neuroinflammatory response seems to best represent GWI with past traumatic events (i.e., GWI with probable PTSD; GWI_H_), while the animal model without corticosterone priming best represents GWI alone (i.e., GWI without probable PTSD; GWI_L_). However, while the animal models at rest topologically resemble the GWI_H_ and GWI_L_ states, the challenged condition in human and nonhuman animals is more closely aligned and represents the best scenario for assessing treatments.

By applying our genetic algorithm methodology to these starting conditions, we identify three possible candidate treatment courses for GWI. The first, involving increasing testosterone, followed by inhibiting IL-2, IFNγ, TNFα, or TNFβ, followed by inhibiting the glucocorticoid receptor, applies to both the GWI_L_ and GWI_H_ subgroups and is the only predicted treatment course for GWI_L_ based on this model. This treatment course is consistent with past findings regarding Th1 immunity and glucocorticoids in GWI and our previously predicted treatment course for GWI involving modulating Th1 immunity via inhibiting TNFα and TNFβ via etanercept, followed by blocking the glucocorticoid receptor with mifepristone [65,66], except for the addition of the testosterone pre-treatment. 

There are many anecdotal reports that male veterans with GWI suffer from low levels of testosterone; however, there is a lack of formal studies investigating this claim. Studies of self-reported sexual dysfunction have included reports of decreased libido, erectile dysfunction, discomfort or pain during intercourse, and a burning sensation after sex. A primary study on sexual dysfunction in a Danish cohort of Gulf War veterans found that these sexual problems were associated with traumatic events and not related to differences in major reproductive hormone parameters [67,68]. However, these studies concern Gulf War veterans, which who do not necessarily meet the criteria for Gulf War Illness. Furthermore, Danish troops were in the Persian Gulf region after the war as peacekeepers and were not exposed to sarin [69]. Other studies investigating the testosterone levels in Gulf War veterans compare groups with high or low exposure to depleted uranium. These studies have similarly found no statistically significant differences between these groups in endocrine function (i.e., including serum follicle-stimulating hormone, luteinizing hormone, prolactin, and total testosterone) [70,71,72,73,74,75,76]. This, however, does not rule out the possibility of low testosterone in veterans with Gulf War Illness compared to sedentary Gulf War-era veteran controls. Several studies in rats have identified anti-androgenic effects (i.e., reduced levels of testosterone, follicle-stimulating hormone, and luteinizing hormone, as well as testicular shrinkage) of the GW-relevant organophosphate acetylcholinesterase inhibitor and pesticide, chlorpyrifos [77,78,79,80,81]. Studies investigating the diffuse chronic pain of fibromyalgia, which has commonalities with other chronic pain conditions such as PTSD and Gulf War Illness, have suggested low or deficient testosterone serum levels that likely cause an inflamed nociceptive nervous system [82]. This hypothesis is supported by the relief of chronic pain symptoms following the application of testosterone gel [83]. Therefore, while our modeling efforts indicate the potential for low testosterone in GWI [38,39,66,84], which is supported by trends in our data, there is a lack of conclusive findings in this area; thus, a more formal analysis of endocrine dysfunction in GWI accounting for confounding factors, such as age and BMI, is needed.

Beyond the first three-intervention treatment course predicted for GWI in total, our modeling efforts also identified two additional treatment courses for GWI_H_, each with its separate advantages and disadvantages. The second course is another three-intervention treatment strategy that involves a continuous inhibition of IL-6, which contains an inhibition of IL-2, IFNγ, TNFα, or TNFβ, followed by inhibition of the glucocorticoid receptor with no overlap. Like the addition of testosterone suggested for the first treatment course, this treatment course is consistent with the previously reported treatment course of modulation of Th1 immunity followed by blocking the glucocorticoid receptor, except here it involves the addition of a continual inhibition of IL-6 during the treatment course. Here, the potential drawback is both the continual administration of a potent IL-6 immune inhibitor over the treatment course in addition to another interspersed IL-2, IFNγ, TNFα, or TNFβ immune inhibitor. The advantage of this three-intervention treatment is the suggested doubling of the overall return to health rate compared to the first.

Finally, the third treatment course is a two-intervention strategy involving simultaneous inhibition of IL-6 and the glucocorticoid receptor. This is a novel treatment course for GWI_H_ of minimal intervention with a comparable return to the health rate of the first treatment course. It does involve the continual administration of a potent IL-6 immune inhibitor over the treatment course in conjunction with glucocorticoid inhibition but does not have the same drawback as the second treatment course involving simultaneous dual immune inhibition. 

The inhibition of IL-6 for the treatment of GWI with probable PTSD makes sense in the light of the increased levels of IL-6 found in PTSD. For example, elevated serum IL-6 and IL-6 receptor concentrations have been found in PTSD following accidental man-made traumatic events [85], and enhanced IL-6 response to the mental stress following myocardial infarction [86]. A meta-analysis and meta-regression of 8057 studies comparing inflammatory markers between patients with PTSD and healthy controls found increased levels of IL-6 that are more pronounced in PTSD without major depressive disorder and for those not taking medication, with the severity of PTSD being positively associated with IL-6 levels [87]. Many animal models have also been used to understand how the brain responds to trauma and investigate treatments that can reduce symptoms of PTSD. Despite the varied methodologies used to model PTSD, rodent models have consistently shown elevated inflammatory markers, including IL-6, both in the central nervous system and peripherally after exposure to inescapable shock [88], chronic variable stress [89,90], and social defeat [91]. This indicates that IL-6 is a feature of PTSD-related pathology both in human patients and in rodent models. Interestingly, treatments that alleviate PTSD-related symptoms also reduce IL-6. This finding is corroborated with anti-inflammatory drugs such as minocycline, which reduces IL-6 and anxiety-related behaviors in rodent models of PTSD [92,93]. Not only do these anti-inflammatory drugs have anxiolytic effects, indicating that reducing pro-inflammatory cytokines could ameliorate PTSD, but also the frontline drugs used to treat PTSD, serotonin selective reuptake inhibitors (SSRIs), reduce inflammatory markers such as IL-6 [92,94]. Taken together, trauma that induces PTSD-like features in nonhuman animals increase IL-6 and treatments that reduce anxiety after trauma also reduce IL-6, so it follows that IL-6 may play a role in the mechanism of PTSD, but most data supporting this role of IL-6 are indirect. More recently, inhibiting IL-6 using antibodies or transgenic silencing showed that IL-6 plays a causal role in the enhanced fear memory commonly seen in PTSD [95]. Thus, animal models of PTSD indicate that inflammatory markers, including IL-6, are both a feature and a mechanism underlying PTSD. 

## 4. Materials and Methods

### 4.1. Discrete Ternary Logical Analysis

The discrete ternary logical network analysis used in the present work is an extension of a methodology proposed by Mendoza and Xenarios [40] and Thomas [43], and has been reported previously by our group [38,39,53,66]. We encode documented feedback mechanisms within the endocrine-immune system using only the direction (source and target) and type (activator or inhibitor) of interaction. As data describing the magnitude of changes remains limited, we consider all cell types to be equally responsive to the actions of the cytokines for which they express receptors. Accordingly, we also consider cytokine synthesis to be equivalent regardless of cell type. Using this formalism, we determine the number and type of stable resting states supported by the regulatory circuitry as well as the specific qualitative endocrine-immune signatures at each of these stable points without requiring detailed kinetic information; that is, we determine where the system would eventually come to rest even though we may not know how quickly this equilibrium will be reached. 

In this model, signaling molecules and cell types are represented as individual variables, each capable of adopting three discrete states: −1 (downregulated), 0 (nominal), and 1 (upregulated). At any point in time *t*, the state of a system with *n* variables can be represented by the vector $\vec{x}\left(t\right)$, such that

(1) $$\vec{x}\left(t\right)=\left(x_{1}\left(t\right),\;x_{2}\left(t\right),\cdots ,x_{N}\left(t\right)\right),$$

where $x_{i}\left(t\right)$ is the state of the *i^th^* variable of the *n* variable system at time *t*. The image vector $\vec{x}\left(t+1\right)$ describes the preferred state towards which the system evolves in the next time increment. The state value of the image vector for the *i^th^* variable is determined from its current state and a set of balanced ternary logic statements based on the current value of variable and the mode of action (i.e., activate or inhibit) of the neighboring input variables. These logic statements are expressed as follows (Equation (2)):

(2) $$x_{i}\left(t+1\right)=\{\begin{array}{c} \left(x_{i1}^{A}\left(t\right)\vee x_{i2}^{A}\left(t\right)\vee \cdots \vee x_{iX}^{A}\left(t\right)\right)\nabla \left(x_{i1}^{I}\left(t\right)\vee x_{i2}^{I}\left(t\right)\vee \cdots \vee x_{iY}^{I}\left(t\right)\right)\\ \left(x_{i1}^{A}\left(t\right)\vee x_{i2}^{A}\left(t\right)\vee \cdots \vee x_{iX}^{A}\left(t\right)\right)\\ \neg \left(x_{i1}^{I}\left(t\right)\vee x_{i2}^{I}\left(t\right)\vee \cdots \vee x_{iY}^{I}\left(t\right)\right) \end{array},$$

where the ∇, ∨, and ¬ symbols are ternary HIGH/LOW PASS, OR and NOT operators, $x_{ij}^{A}$ is the state of the *i^th^* variable’s *j^th^* activator, and $x_{ij}^{I}$ is the state of the *i^th^* variable’s *k^th^* inhibitor. The ternary operators given in Equation (2) are described in further detail in [38]. The first entry in Equation (2) is used when the variable possesses *X* activators and *Y* inhibitors, the middle when the variable has only *X* activators, and last when the activator has only *Y* inhibitors. While the number of *X* activators and/or *Y* inhibitors for a given variable may remain static, they may also be allowed to change based on predefined conditions, such as the state of one or more variables. In the case where the number of *X* activators and/or *Y* inhibitors are conditionally dependent, Equation (2) is modified to reflect the number of activators or inhibitors allowed by the predefined conditions and the current state of the network system. In this case of this model conditional edges are only allowed if the EndothelialCells node is −1.

### 4.2. Monte Carlo Simulation of State Evolution 

Following [38], the evolution of state transitions supported by the model was analyzed by developing a Monte Carlo simulation algorithm. From any initial starting state, allowable state transitions are determined based on Equation (2). Applying Equation (2) to each variable in the model for the state of the system at time *t*, $\vec{x}\left(t\right)$ defines the image vector $\vec{x}\left(t+1\right)$ for the next timepoint. With $\vec{x}\left(t+1\right)$ defined, the system is updated asynchronously allowing only one variable to change at each timestep following the generalized logical analysis of Thomas [43]. According to this method, one variable slated to change by the image vector is chosen at random using a uniform equal distribution and used to generate the next allowable state; that is, if the *i^th^* variable of the state vector, $x_{i}\left(t\right)$, is chosen at random to evolve it is moved one step towards its preferred image $x_{i}\left(t+1\right)$ (e.g., if $x_{i}\left(t\right)=-1$ and $x_{i}\left(t+1\right)=1$, then $x_{i}\left(t+1\right)$ is set to 0). Thus, for each current state of the system there are potentially several subsequent states towards which it may asynchronously evolve. States for which the image vector is the same as the current state vector are considered stable (steady states, attractors, basins, etc.), and do not evolve further in time. The Monte Carlo procedure is performed until such a stable state is reached. Executing the simulation multiple times gives a distribution of paths that is used to determine the behavior of the system from any given start state. 

### 4.3. Participants/Procedure

All participants signed an informed consent approved by the Institutional Review Board of the Miami Veterans Affairs Medical Center. Ethics review and approval for data analysis was also obtained by the IRB of Nova Southeastern University.

Participants were recruited via the Miami Veterans Affairs (VA) Medical Health Center in two cohorts funded under a Veterans Affairs Merit award (GWI: *n*  =  27, healthy controls (HC): *n*  =  19) and a Department of Defense Gulf War Illness Research Program award (W81XWH-09-2-0071) (GWI: *n*  =  24, HC: *n*  =  19), both of which compared male veterans with GWI to HC. Therefore, the full sample for the multivariate and univariate analyses included *n*  =  51 male veterans with GWI in addition to *n*  =  38 healthy controls. 

Inclusion criteria for GWI participants was derived from Fukuda et al. [96] and consisted in identifying veterans deployed to the theater of operations between 8 August 1990, and 31 July 1991, with one or more symptoms present for 6 months from at least 2 of the following: fatigue, mood, and cognitive complaints, as well as musculoskeletal complaints. Participants were in good health prior to 1990 and had no current exclusionary diagnoses defined by Reeves et al. [97]. This includes exclusion of major dementias of any type and alcoholism or drug abuse, medical conditions including organ failure, rheumatologic disorders, and use of medications that impact immune function, such as steroids or immunosuppressants. Collins et al. [98] supports the use of the Fukuda definition in GWI. Control participants consisted of Gulf War era veterans self-defined as healthy with no exclusionary diagnoses, and sedentary (no regular exercise program, sedentary employment).

All participants were subjected to a standard maximal graded exercise test to stimulate their immune response and blood samples were collected at rest (T0) and peak effort (T1) as previously described [6]. 

### 4.4. Human Blood Analysis

Plasma was collected immediately upon receiving. EDTA Plasma was collected by centrifugation, 2000× *g* for 15 min, and frozen immediately. Cytokine analysis was performed using Quansys chemiluminescent assays (Quansys Biosciences, Logan, Utah). The Quansys Imager, driven by an 8.4-megapixel Canon 20D digital SLR camera, supports 96-well plate-based chemiluminescent imaging. The Q-Plex™ Human Cytokine Screen is a quantitative ELISA-based test where distinct capture antibodies have been absorbed to each well of a 96-well plate in a defined array. Custom 16- and 18-multiplex assays were used in the Veterans Affairs Merit award and a Department of Defense Gulf War Illness Research Program award (W81XWH-09-2-0071) studies, respectively.

The 16-multiplex panel includes TNFα, TNFβ, IL-1α, IL-1β, IL-6, IFN-γ, IL-12, IL-2, IL-15, IL-8, IL-5, IL-17, IL-23, IL-10, and IL-13 (Fletcher et al. (2009)). For the standard curves, we used the second order (k = 2) polynomial regression model (parabolic curve), Y = b_0_ + b_1_X + b_2 ×_^2^.... + b_k_X^k^, where Y caret is the predicted outcome value for the polynomial model with regression coefficients b_1_ to k for each degree and y intercept b_0_. Quadruplicate determinations were made, i.e., each sample was run in duplicate in two separate assays.

The 18-multiplex panel includes TNFα, TNFβ, IL-1α, IL-1β, IL-6, TNF-RI, TNF-RII, IFN-γ, IL-12, IL-2, IL-15, IL-8, IL-5, IL-17, IL-23, IL-10, and IL-13. Briefly, plasma samples are thawed at 4 °C overnight. Samples are plated in duplicate following the manufacturer’s protocol. The plates were read at 270 s using the Q-view Imager LS (Quansys). Individual cytokine concentrations were obtained using image analysis software (Q-view, Quansys Biosciences). Sample concentrations were calculated from standard curves created by a five-parameter logistic regression (5PL) with √y weighting. The average value from each duplicate was then used for subsequent analyses.

To account for 16-plex and 18-plex differences, data were separated and standardized by z-scoring. The two data sets were then combined and reverse z-scored using the mean and standard deviation of the 18-plex dataset to scale the 16-plex data to the 18-plex set. Following this, the dataset was normalized using min–max normalization so the final data values ranged between 0 and 1. Normalized cytokine values are given in Table S1.

### 4.5. Animals and Dosing

All animal experiments were performed using protocols approved by the Centers for Disease Control—National Institute for Occupational Safety and Health Institutional Animal Care and Use Committee (CDC-Morgantown IACUC) and the United States Army Medical Research and Development Command Animal Care and Use Review Office (USAMRDC ACURO). Adult male C57BL/6J mice (8–12 weeks of age; weighing approximately 22 g) were purchased from Jackson Labs (Bar Harbor, ME, USA). Mice were single housed in a temperature- (21 ± 1 °C) and humidity-controlled (50 ± 10%) room under filtered positive-pressure filtration and a 12 h light/dark cycle (lights on at 0600 EDT); mice were given ad libitum access to food (Harlan 7913 irradiated NIH-31 modified 6% rodent chow) and water.

For dosing, mice were randomly placed into groups (*n* = 5/group) and either remained on regular drinking water or pretreated with CORT (Steraloids Inc., Newport, RI, USA) in the drinking water (200 mg/L in 0.6% Ethanol (EtOH; Sigma-Aldrich, Inc., St. Louis, MO, USA)) ad libitum for 4 days followed by exposure to DFP (4 mg/kg, i.p.; Sigma-Aldrich, Inc., St. Louis, MO, USA) or saline(0.9%) on the 5th day. Following this exposure combination, modeling the initiating factors for GWI [50,51,54,61], mice were repeatedly exposed to 4-day bouts of CORT every other week (4 days on/10 days off) for a total of 11 weeks. Following the final 4-day CORT exposure, mice were challenged with the inflammatory bacterial endotoxin lipopolysaccharide (LPS; 0.5 mg/kg, s.c.; Sigma-Aldrich, Inc., St. Louis, MO, USA) or saline and sacrificed by decapitation at 6 h following this exposure. Frontal cortex and blood serum samples were collected as previously described [50,51,54,61]. Due to mortality associated with DFP exposure, the DFP and DFP LPS groups comprised an *n* of 2 and 3, respectively. A schematic of the dosing paradigm is shown in Figure 2A.

### 4.6. Mouse Serum Cytokine Analysis

Cytokine levels were measured in mouse serum (*n* = 2–5/group) as previously described [61]. Briefly, serum samples were analyzed using Q-Plex™ Mouse Cytokine Screen (16-plex), Quansys Imager, and Quansys reagents (Quansys Biosciences, Logan, Utah). Cytokine levels are expressed as pg/mL and presented as the mean ± SEM in Supplementary Table S2.

### 4.7. qRT-PCR Analysis of Mouse Brain Cytokines

mRNA was isolated and cytokine expression measured by qRT-PCR analysis as previously described [50,99]. Briefly, total RNA was isolated from the frontal cortex using Trizol Reagent (Thermo Fisher Scientific, Waltham, MA, USA), Phase-lock heavy gel (Eppendorf AG, Hamburg, Germany), and RNeasy mini spin columns (Qiagen, Valencia, CA, USA) following the manufacturer’s instructions. PCR analysis of the housekeeping gene, glyceraldehyde-3-phosphate dehydrogenase (GAPDH), and of the proinflammatory mediators, TNFα, IL-6, CCL2, IL-1β, leukemia inhibitor factor (LIF), and oncostatin M (OSM) was performed in an ABI7500 Real-Time PCR System (Thermo Fisher Scientific) in combination with TaqMan^®^ chemistry. Relative quantification of gene expression was performed using the comparative threshold (ΔΔC_T_) method to normalize expression changes against the GAPDH control, as well as normalize the expression changes to the corresponding saline-treated controls (Figure 2C, and Table S3). Statistical significance (*p* ≤ 0.05) was determined by two-way ANOVA followed by multiple comparisons using Fisher’s Least Significant Difference in SigmaPlot (v. 14, Systat Software, Inc., San Jose, CA, USA).

### 4.8. Comparison of the Gene Expression Data to the Model-Predicted States

Following [38], to check the validity of our model, we compared stable states predicted by the latter to gene expression profiles measured experimentally in the mouse model of Gulf War Illness [51] in both brain and blood, and to blood measures using the cohort of male veterans. Brown’s theoretical approximation [100] of Fisher’s statistics was used as a measure of similarity between a given model-predicted state and the expression profile measured in a subset of genes corresponding to the cellular and molecular entities used in our model, as done in our previous work [38,39,66,84]. Fisher’s statistics provide a meta-analysis technique to combine probabilities and obtain the overall significance of a set of *p*-values corresponding to independent tests of the same null hypothesis. The combined *χ*^2^ statistic,

(3) $$T_{0}=-2\overset{n}{\underset{i\;=1}{\sum }}\ln\left(p_{i}\right)$$

where *n* is the number of measurable variables and *p*_i_ are the corresponding *p*-values under the null hypothesis, has a *χ*^2^ distribution with *2N* degrees of freedom assuming the hypothesis tests are independent. As evidenced by the connectivity of the system studied here, these model entities do not express independently. As a result, direct application of this test statistic is not valid since the assumption of independence is violated. To accommodate this, Brown [100] suggested a method for combining non-independent tests. If the tests are not independent, then the statistic *T*_0_ has mean *m* *=* 2*N* and variance (*σ*^2^), given as

(4) $$\sigma^{2}=4N+2\sum_{i=1}^{N-1}\sum_{j=i+1}^{N}cov\left(-2lnp_{i},-2lnp_{j}\right)$$

where *p_i_* and *p_j_* are the *p*-values for each test and the covariance (*cov*) is calculated as

(5) $$cov\left(-2lnp_{i},-2lnp_{j}\right)=\{\begin{array}{cc} \rho_{ij}\left(3.25+0.75\rho_{ij}\right), & 0\leq \rho_{ij}\leq 1\\ \rho_{ij}\left(3.27+0.71\rho_{ij}\right), & -0.5\leq \rho_{ij}<0 \end{array}$$

with *ρ_ij_* being the unadulterated correlation between variable *i* and variable *j*. Finally, the overall significance *p* of a set of non-independent tests is calculated using the statistic *T* which under the null hypothesis follows the central *χ*^2^ distribution, where *T = T*_0_*/c* with *2N/c* degrees of freedom and *c =* *σ*^2^*/*4*N*.

Here, we test if the expression levels of the subset of genes corresponding to the cellular and molecular entities used in our model align with a given model-predicted discrete state profile. Our null hypothesis is that the experimental measures do not align with model predictions of greater than control (normal), lower than the control or in alignment with the control levels. First, the *p*-values for individual variables, *p_i_*, were calculated using two-sample *t*-tests comparing the expression levels in ill subjects with those measured in healthy controls. Where the model predicts marker expression to be high (+1), a ‘right-handed’ one-tailed test was used to confirm that the measured expression levels are significantly greater than the reference control (0). Conversely, a ‘left-handed’ test was used when the model predicted a low (−1), to confirm that the measured expression levels are on average significantly lower than the reference control (0). For the case where the model predicted normal expression levels for a variable (0), a two-tailed *t*-test was used. However, the *p*-value from the two-tailed test, *p_two-tail_*, gives the probability that there is an observable difference between illness and control, which is the null hypothesis. To rectify this, when comparing to a model-predicted variable of 0 we took the *p*-value to be *p_i_ = 1 − p_two-tail_*, giving the probability of obtaining the predicted value when the null hypothesis is true. The unadulterated correlation values *ρ_ij_* between two variables *i* and *j* were calculated in the control groups as the pairwise Pearson’s linear correlation coefficient between variables. Aggregate *p*-values between the predicted model states were determined via Brown’s method above, where values between the predicted states that were found to disagree with the *p*-values for individual variables, *p_i_*, were taken as 1. Conversely, when the values between the predicted states were found to agree, *p_i_* was assigned a standard minimum value for significance of 0.05 to avoid numerical instability in the calculation of the combined *χ*^2^ statistic *T*_0_. Hold-out cross validation was performed by randomly splitting the healthy control dataset evenly in two and performing the above method 100 times. This resulted in a maximum of 94% of the runs, finding that the test group aligned with the healthy SS0 stable state with an even distribution of the remaining cases being assigned to the other alternate stable states (see Figure S1).

A comparison of the measured states with the model-predicted stable states is best visualized by projecting the multi-dimensional co-expression profiles into a two-dimensional space using multidimensional scaling. Here, the dissimilarity matrix defined by the *p*-value is scaled such that the 2D Euclidean distances between points approximate of the corresponding dissimilarities. This is performed using the function *mdscale* in MATLAB to minimize Kruskal’s stress criterion normalized by the sum of squares of the dissimilarities. To maintain the relative structure of the healthy control baseline-defined stable state positions between the conditions, the weight of the added illness condition distances was set at ten percent of the distances defining the stable state positions. This value was chosen to keep the RMSD of the stable states between conditions equivalent to two decimal places. The resulting 2D plot illustrates the statistical significance of separation between the measured and predicted co-expression patterns. To compare the plots between the conditions, all the resulting 2D plots were aligned with the human baseline condition via an affine geometric transformation based on the model-predicted stable state positions via the MATLAB function *fitgeotrans*. 

To provide a measure of agreement between conditions, the root mean square deviation (RMSD) between the aligned 2D plots was calculated as

(6) $$RMSD=\sqrt[{2}]{\frac{1}{N}\overset{N}{\underset{i=1}{\sum }}{\left(x_{i}^{B}-x_{i}^{A}\right)}^{2}+{\left(y_{i}^{B}-y_{i}^{A}\right)}^{2}}$$

where $x_{i}^{j}$ and $y_{i}^{j}$ are the horizontal and vertical positions of the *i^th^* point of *n* total points in plot *j*. 

### 4.9. Simulating Intervention Courses

To identify a robust sequence of interventions capable of moving the system from a pathological mode of regulation to that of normal health, we evolved solutions combining a specific choice of treatment targets as well as the sequence, spacing, and type of external perturbation. For each of these candidate treatment courses, simulations were conducted to evaluate the occurrence of normal homeostasis. Specifically, each clinical intervention was represented as a treatment vector with *n* variables. Interventions applied to the system state at some point in time, *t*, are represented by the vector $\vec{T}\left(t\right)$, such that

(7) $$\vec{T}\left(t\right)=\left(T_{1}\left(t\right),T_{2}\left(t\right),\cdots ,T_{N}\left(t\right)\right)$$

where $T_{i}$ is a ternary value describing the effect of the clinical treatment on the *i^th^* element of the system: −1 (inhibiting), 0 (untreated), and 1 (supplementing). At those times where an intervention is being applied, the image vector $\vec{x}\left(t+1\right)$ describing the preferred state towards which the system should evolve is now defined as

(8) $$\vec{x}\left(t+1\right)\;=\;\vec{x}\left(t\right)+\vec{T}\left(t\right)$$

as opposed to the unperturbed logic described in Equation (2). Due to the ternary nature of this system no value can extend beyond the range of −1 to 1; hence, values beyond this range were rounded accordingly (i.e., if $x_{i}\left(t\right)=1$ and $T_{i}\left(t\right)=1$, then $x_{i}\left(t+1\right)=\;x_{i}\left(t\right)+T\left(t\right)=\;2$ is rounded to 1). At times *t* when there is no treatment applied (i.e., all T(t)=0), the state transition continues according the logic in Equation (2).

### 4.10. Genetic Algorithm for Optimizing Treatment Course 

A first mapping of the illness basin of attraction consisted of a series of simulations where we first allowed for a one-time simultaneous perturbation of two or more variables at the outset only without subsequent intervention. We then expanded on this by performing a global search to find an optimal series of single target interventions separated in time, which reliably led to health. A Genetic Algorithm (GA)-based search [66] was used to optimize this treatment course, as its form naturally accommodates the discrete definition of each system state. A treatment course vector $\vec{C}$ with *M* interventions is therefore defined as

(9) $$\vec{C}\;=\;\left(\vec{T_{1}}\left(t_{1start},t_{1stop}\right),\vec{T_{2}}\left(t_{2start},t_{2stop}\right),\cdots ,\vec{T_{M}}\left(t_{Mstart},t_{Mstop}\right)\right)$$

where ${\vec{T}}_{j}\left(t_{istart},t_{istop}\right)$ is the *j^th^* intervention treatment vector starting at time point $t_{jstart}$ and ending at $t_{jstop}$. Due to the asynchronous nature of the model each treatment vector $\vec{T}$ only contains a single target intervention $T_{i}$ that affects the *i^th^* element variable in the system at any given time step. 

The GA starts by generating a population of 1000 candidate treatment courses each composed of a specific number of randomly selected interventions applied at random time points. The response of the system to each treatment course in this initial generation of candidates is then simulated for 1000 timesteps. Over the course of these time steps the state of the system evolves according to Equation (2), except at those times when interventions are applied. At these intervention events the state transition follows Equation (5). These 1000 iterations provide a distribution of paths that are then ranked according to a fitness function based on the number of times a treatment successfully reaches the healthy stable state (% HHM). After all treatments in the generation have been ranked, the top 10% are retained without change for the next generation. Thus, treatments generated by the GA that have a higher probability of leading to the HHM are re-executed and re-evaluated over many generations. The remainder of the next generation of candidate solutions is created by choosing random pairs from the total set of treatment courses (including the top 10*^th^* percentile) and combining (cross-over recombination) them. Combination, ⊗, of two treatment courses ${\vec{C}}_{1}$, and ${\vec{C}}_{2}$ is performed at a single random splice timepoint *s* to create a new treatment course ${\vec{C}}_{1}^{\prime }$ for the next generation, such that all treatments in ${\vec{C}}_{1}$ preceeding *s*, and all treatments in ${\vec{C}}_{2}$ after *s* are included in ${\vec{C}}_{1}^{\prime }$. 

The response to the new treatment courses, including members of the previous top 10th percentile, are then simulated once again and ranked as a new generation. This process is continued iteratively for 1000 generations. The final treatment course with the highest % HHM was taken as the best treatment solution for a given run. The overall best treatment course was chosen from the 100 repeated GA runs.

## 5. Conclusions

Our simulations predict treatment avenues for GWI in totality, as well as specific treatments for GWI with probable PTSD. However, it must be noted that the predictions presented here are based on an idealized model of immune interactions across the BBB. The alignment of experimental data with model predictions does suggest that GWI aligns with a state characterized by BBB dysfunction as predicted by the model; however, additional molecular and biochemical evidence (e.g., small molecule leakage, albumin leakage, MMP activation, and monocyte adhesion to the microvascular endothelium) are needed to make a definitive claim concerning BBB function in GWI and GWI mouse models. As such, future research in this area is warranted. Overall, the advantage of this modeling methodology is also one of its limitations in that it does not account for detailed kinetics. The magnitude and transition time of the interactions between elements of the extended neuroendocrine-immune system are not predicted, rather only the logical feasibility of these interventions is assessed. While refinement of the simulation parameters based on detailed measurement of the model elements can serve to improve the accuracy of the simulation timing and magnitude, the underlying logic is not affected. Regardless, even with these refinements, the safety and efficacy of any predicted treatment strategy must be determined clinically. 

## Figures and Tables

**Table 1:** GWI cohort demographics.

Group	Total	GWI	GWI_H_	GWI_L_	HC	p_3_	p_2_
***n***	89	51	32	19	38		
**Mean Age (y)**	43.7 ± 7.4	43.8 ± 5.7	44.4 ± 5.0	42.8 ± 6.7	43.5 ± 9.3	0.991	0.979
**Mean BMI**	29.5 ± 5.4	30.3 ± 4.6	30.3 ± 4.1	30.2 ± 5.4	28.5 ± 5.4	0.958	0.803
**Race**						0.318	0.245
*Asian*	2.2%	3.9%	3.1%	5.3%	0.0%		
*Black*	22.5%	27.5%	34.4%	15.8%	15.8%		
*Hispanic*	46.1%	39.2%	40.6%	36.8%	55.3%		
*White*	28.1%	29.4%	21.9%	42.1%	26.3%		
*Other*	1.1%	0.0%	0.0%	0.0%	2.6%		
**Marital Status**						0.755	0.838
*Married*	62.9%	64.7%	71.9%	52.6%	60.5%		
*Widowed*	1.1%	0.0%	0.0%	0.0%	2.6%		
*Divorced*	12.4%	13.7%	12.5%	15.8%	10.5%		
*Separated*	3.4%	3.9%	3.1%	5.3%	2.6%		
*Never Married*	9.0%	7.8%	9.4%	5.3%	10.5%		
*Not Answered*	11.2%	9.8%	3.1%	21.1%	13.2%		
**Employed**	75.3%	70.6%	68.8%	73.7%	81.6%	0.456	0.234
**Mean Schooling (y)**	15.5 ± 2.5	15.3 ± 2.6	15.7 ± 2.8	15.4 ± 2.0	15.8 ± 2.8	0.994	0.907

**Table 2:** The three intervention treatments course starting from SS5 results in a 37% return to health.

Treatment	t_start_	t_stop_
Increase Testosterone	1	43
Inhibit CK1	21	83
Inhibit GRD	75	99

**Table 3:** The three intervention treatments course starting from SS4 results in a 61% return to health.

Treatment	t_start_	t_stop_
Inhibit MK6	1	100
Inhibit CK1	8	40
Inhibit GRD	77	99

**Table 4:** The two intervention treatments course starting from SS2 results in a 31% return to health.

Treatment	t_start_	t_stop_
Inhibit MK6	1	96
Inhibit GRD	7	93

## Data Availability

Data supporting the reported results can be found in the Supplementary Materials provided. PCR data from the GWI mouse model study is available on the NIOSH Data and Statistics Gateway (https://www.cdc.gov/niosh/data).

## Supplementary Materials

The following are available online at https://www.mdpi.com/article/10.3390/ijms22168546/s1.
