# Supplementary material for: Modeling Neuroimmune Interactions in Human Subjects and Animal Models to Predict Subtype-Specific Multidrug Treatments for Gulf War Illness
Source: Int J Mol Sci. 2021 Aug 9;22(16):8546. doi: 10.3390/ijms22168546 (PMC8395153; doi:10.3390/ijms22168546)
Supplement: Supplementary file 1 [file ijms-22-08546-s001.zip › ijms-1318603-supplementary.pdf]

Modeling Neuroimmune Interactions in Human Subjects and Animal Models to Predict Subtype Specific Multidrug Treatments for Gulf War Illness

Francisco J. C. Arias, Kristina Aenlle, Maria Abreu, Mary A. Holschbach, Lindsay T. Michalovicz, Kimberly A. Kelly, Nancy Klimas, James O'Callaghan, Travis J. A. Craddock

Supplementary Information

Table S1. Human plasma cytokine levels normalized mean ± SEM

Group	IL-1a	IL-1b	IL-2	IL-4	IL-5	IL-6	IL-8	IL-10	IL-12	IFNy	TNFa
						Mean					
HC	0.133	0.134	0.076	0.207	0.075	0.117	0.039	0.152	0.103	0.230	0.111
GWIL T0	0.096	0.083	0.088	0.200	0.084	0.194	0.085	0.103	0.065	0.249	0.088
GWIH T0	0.109	0.150	0.052	0.184	0.042	0.089	0.097	0.077	0.065	0.260	0.136
GWIL T1	0.084	0.198	0.083	0.125	0.066	0.159	0.253	0.114	0.161	0.321	0.187
GWIH T1	0.107	0.296	0.056	0.137	0.064	0.135	0.255	0.110	0.127	0.323	0.208
						SEM					
HC	0.030	0.025	0.028	0.032	0.028	0.027	0.005	0.037	0.035	0.017	0.024
GWIL T0	0.021	0.012	0.036	0.036	0.042	0.055	0.036	0.035	0.016	0.021	0.014
GWIH T0	0.018	0.042	0.009	0.026	0.006	0.011	0.030	0.015	0.012	0.034	0.028
GWIL T1	0.014	0.047	0.043	0.009	0.042	0.051	0.048	0.045	0.048	0.036	0.024
GWIH T1	0.032	0.051	0.013	0.019	0.018	0.037	0.034	0.019	0.017	0.039	0.036

Table S2. Mouse serum cytokine levels in pg/mL ± SEM.

Group	mIFNy	mIL-1a	mIL-1b	mIL-2	mIL-4	mIL-5
Water Saline	9.98 ± 1.15	11.16 ± 2.76	29.99 ± 1.74	14.53 ± 1.18	7.98 ± 1.00	35.68 ± 2.77
Water DFP Saline	14.21 ± 6.69	11.79 ± 4.41	25.04 ± 0.24	15.66 ± 2.26	10.32 ± 0.33	31.75 ± 0.07
Water DFP LPS	9.76 ± 1.37	33.84 ± 14.38	44.91 ± 6.93	18.04 ± 1.48	8.14 ± 0.18	40.15 ± 2.76
CORT DFP Saline	10.43 ± 0.63	2.18 ± 0.04	29.86 ± 3.03	16.49 ± 1.82	7.82 ± 0.60	28.55 ± 0.52
CORT DFP LPS	24.80 ± 3.16	96.38 ± 22.48	185.42 ± 30.52	25.28 ± 5.73	11.12 ± 2.24	99.58 ± 18.00

Group	mIL-6	mIL-10	mIL-12	mKC	mMIP-2	mTNFa
Water Saline	48.25 ± 27.74	3.92 ± 0.10	15.98 ± 1.00	42.14 ± 23.02	15.02 ± 0.68	3.04 ± 0.14
Water DFP Saline	18.09 ± 1.84	4.05 ± 0.38	18.22 ± 5.21	43.14 ± 20.25	14.92 ± 0.59	2.68 ± 0.30
Water DFP LPS	997.73 ± 606.52	5.00 ± 0.78	14.20 ± 1.20	418.86 ± 187.08	16.01 ± 0.50	8.92 ± 3.72
CORT DFP Saline	22.04 ± 3.36	3.87 ± 0.13	16.52 ± 1.29	39.25 ± 11.72	15.41 ± 0.87	3.51 ± 0.55
CORT DFP LPS	9901.16 ± 1796.02	44.68 ± 6.73	16.38 ± 0.83	1971.15 ± 280.62	97.32 ± 17.56	38.79 ± 5.72

Table S3. Mouse cortex cytokine mRNA normalize expression changes against the GAPDH ± SEM.

Group	CCL2	IL6	OSM	TNFa	LIF	IL1b
Water Saline Saline	1.05 +/- 0.16	1.00 +/- 0.04	1.00 +/- 0.02	1.01 +/- 0.07	1.00 +/- 0.06	1.02 +/- 0.10
Water DFP Saline	0.89 +/- 0.01	1.00 +/- 0.15	1.18 +/- 0.02	0.86 +/- 0.33	0.65 +/- 0.28	1.14 +/- 0.06
Water DFP LPS	2.07 +/- 1.17	0.79 +/- 0.23	1.55 +/- 0.50	5.58 +/- 2.54	0.53 +/- 0.15	5.00 +/- 3.09
CORT DFP Saline	0.69 +/- 0.33	0.38 +/- 0.16	0.90 +/- 0.28	2.10 +/- 1.49	0.22 +/- 0.08	1.23 +/- 0.83
CORT DFP LPS	24.20 +/- 4.03	1.82 +/- 0.40	5.95 +/- 0.61	35.06 +/- 5.60	1.31 +/- 0.55	85.88 +/- 7.26

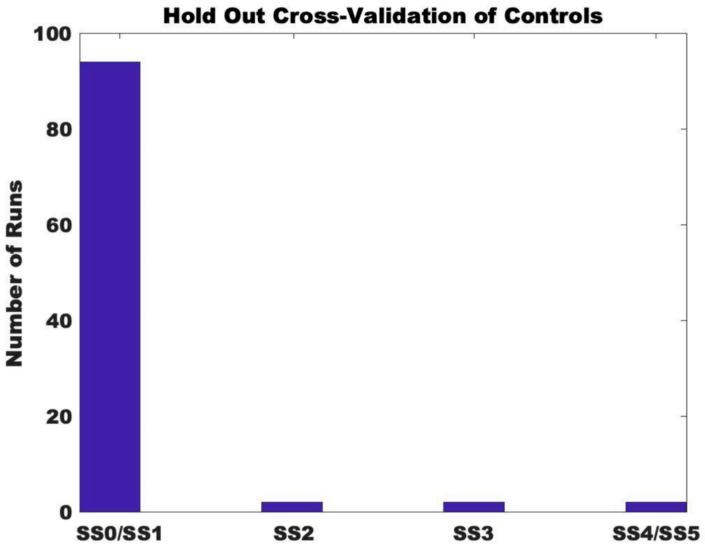

Figure S1: Hold-out cross validation was performed by randomly splitting the healthy control dataset evenly in two using the MATLAB function *cvpartition* then performing Brown's method as described in the main Methods section 4.8 to find the stable state (SS) with the closest alignment. This process was repeated 100 times. This resulted in a maximum of 94% of the runs finding that the test group aligned with the healthy stable state (SS0/SS1) with an even distribution of the remaining cases being assigned to the other alternate stable states.
